# Interaction of Wild Strains of *Aspergilla* with *Aspergillus parasiticus* ATCC15517 and Aflatoxin Production †

**DOI:** 10.3390/ijms9030394

**Published:** 2008-08-12

**Authors:** H. Marina Martins, Inês Almeida, Marta Marques, Fernando Bernardo

**Affiliations:** 1 INRB,I.P.-Laboratório Nacional Investigação Veterinária, Estrada de Benfica, 701, 1549-011, Lisboa, Portugal; E-mail: marina.martins@lniv.min-agricultura.pt; coimbra.mm@gmail.com; 2 Escola Superior de Hotelaria e Turismo do Estoril, Av. Condes Barcelona, 2769-510, Estoril, Portugal; E-mail: lines.almeida@eshte.pt; 3 CIISA- Faculdade Medicina Veterinária, Av. Universidade Técnica de Lisboa, 1300-477, Lisboa, Portugal; E-mail: bernardo@fmv.utl.pt

**Keywords:** Aflatoxins, Micotoxins, Biosynthesis, *Aspergillus parasiticus*, *synergism*

## Abstract

Aflatoxins are secondary metabolites produced by some competent mould strains of *Aspergillus flavus, A. parasiticus* and *A. nomius*. These compounds have been extensively studied with regards to their toxicity for animals and humans; they are able to induce liver cancer and may cause a wide range of adverse effects in living organisms. Aflatoxins are found as natural contaminants of food and feed; the main line of the strategy to control them is based on the prevention of the mould growth in raw vegetable or during its storage and monitoring of each crop batch. Mould growth is conditioned by many ecological factors, including biotic ones. Hazard characterization models for aflatoxins in crops must take into consideration biotic interactions between moulds and their potential effects on growth development. The aim of this work is to study the effect of the biotic interaction of 14 different wild strains of *Aspergilla* (different species), with a competent strain (*Aspergillus parasiticus* ATCC 15517) using an *in vitro* production model. The laboratory model used was a natural matrix (humidified cracked corn), on which each wild strain challenged the aflatoxin production of a producer strain. Cultures were incubated at 28°C for 12 days and sampled at the 8^th^ and 12^th^. Aflatoxin detection and quantification was performed by HPLC using a procedure with a MRPL = 1 μg/kg. Results of those interactive cultures revealed both synergic and antagonistic effects on aflatoxin biosynthesis. Productivity increases were particularly evident on the 8^th^ day of incubation with wild strains of *A. flavipes* (+ 70.4 %), *A. versicolor* (+ 54.9 %) and *A. flavus* 3 (+ 62.6 %). Antagonistic effects were found with *A. niger* (− 69.5%), *A. fumigatus* (− 47.6 %) and *A. terreus* (− 47.6 %) on the 12^th^ day. The increased effects were more evident on the 8^th^ of incubation and the decreases were more patent on the 12^th^ day. Results show that the development of *Aspergilla* strains concomitantly with competent aflatoxin producing moulds has a significant influence on the natural biosynthesis pattern.

## Introduction

Aflatoxins B_1_, B_2_, G_1_, and G_2_ are biotoxins synthesized under appropriate ecological conditions by some competent mould strains belonging to the groups *Aspergillus flavus, A. parasiticus* and *A. nomius*. The ability of competent *Aspergilla* to produce aflatoxins depends on the individual metabolic systems, particularly to the primary metabolism of lipids and specified enzymes (synthetases) able to produce the secondary metabolites [1].

These secondary metabolites have been extensively studied since the earlier sixties, with regards to their toxicity towards animals and humans. Aflatoxins, especially B1, may induce liver cancer and can cause a wide diversity of adverse effects in living organisms, including mutagenic, teratogenic and carcinogenic results [2–4]. The most constant effect is the depression of protein synthesis, including that of antibodies. The severity of the adverse effects is proportionate to the level/doses of the exposure.

Nowadays, aflatoxins are the most frequent hazard referred in food imported into the EC, according to the annual reports of Rapid Alert System for Food and Feed [5]. Aflatoxins are found as natural contaminants of a wide range of foods and feeds: cereals and other crops, dry fruits, and milk (ruminant’s metabolize aflatoxins M1 and M2).

The main strategic line to control aflatoxins is based on the prevention of the mould growth in raw vegetables or during vegetative development, harvest, storage and transportation, through a adequate monitoring system applied to each crop batch. Mould growth is conditioned by many ecological factors, including physical-chemical factors and also biotic ones. Aflatoxin production levels are affected by many abiotic parameters like temperature, water availability, pH, osmotic pressure, oxi/reduction potential and chemical nature of nutrients.

The ecological conditions that are favourable to aflatoxin biosynthesis are also suitable for the growth of all the concomitant moulds that might colonize a specific crop. From this perspective it is important to understand under what condtions these developments of mycobiota may interfere with the normal biosynthesis of aflatoxins (biotic factors). The full knowledge of biosynthesis pathway will only became better understood when interactive and multi-factorial studies are performed. The aim of this work was to study the influence of biotic interactions, using an *in vitro* model, whereby 14 different wild strains of *Aspergilla* challenged an aflatoxin-producing strain of *A. parasiticus* (ATCC 15517).

## Materials and Methods

### Strains

*A. parasiticus* ATCC 15517 (aflatoxin-producing strain) and 14 wild *Aspergilla* strains commonly found in crops (won laboratory collection): *A. candidus, A. clavatus, A. flavipes, A. restritus, A. niger, A. versicolor, A. ochraceus, A. glaucus, A. terreus, A. fumigatus and A. flavus* (five isolates). *A. flavus* isolates were previous tested for their ability to synthesize aflatoxins and revealed negative. Mould colonies were maintained on Czapek agar (OXOID CM. 549), incubated at 25° C for 8 days [3].

### “In vitro” aflatoxin production model

Each culture for aflatoxin production was performed by individually challenging the competent *A. parasiticus* reference strain. Each assay were carried out in four Erlenmeyer flasks, each containing sterilised cracked corn (50 g), and distilled water for an a_*W*_ adjustment to 0.98 [6].

Autoclaved substrate was inoculated separately with spore suspensions:

a) References were inoculated with spore suspension of *A. parasiticus* ATCC 15517 (2 mL), diluted to 50%; spores densities were estimated using an opacity gradient equivalent to 0.5 Macfarland.
b) Interactive cultures of the two strains (*A. parasiticus* ATCC 15517 more each of the other *Aspergilla* isolates) were inoculated with 1 ml of each spore suspension.

The flasks that were inoculated with the reference and with the mixed strains were manually shaken daily during 5 min to obtain an adequate homogeneity. Incubations were performed at 28°C for 8 and 12 days, respectively, for two series of flask cultures [7].

### Extraction and immunoaffinity column chromatography

Extraction and immunoafinity column clean step was performed according to the method described by Stroka and Anklam [8]. The samples were extracted with acetonitrile-water solution (85/15) (V/V). The extracted was filtered and diluted (5 mL) with phosphate buffer saline (PBS, 95 mL). Filtrate was passed through an immunoaffinity column (Afla B G.1003, VICAM) and AFS were eluted with 1.25 mL and washed with water (1.75 mL). Eluate was collected and directly used for the HPLC analysis.

### Aflatoxin quantification (HPLC)

Determination of aflatoxin levels in sample extracts was carried out by isocratic reverse-phase liquid chromatography (HPLC) method using a LiChrospher 100 RP-18 EcoPack column (5 μm, 25 × 4.6 mm i.d., Merck, Portugal), with post-column derivatization involving bromination with pyridinum hydrobromide perbromide (PBPB, Sigma P- 3179, Quimica S.A., Spain) and fluorescence detection (Merck Hitachi, excitation and emission wavelengths were 360 nm and 420 nm, respectively). The mobile phase was water-acetonitrile-methanol solution (6/2/3) (V/V/V), and the flow rates were 1.00 mL/min for mobile phase and 0.30 mL/min for the PBPB reagent. The MRLP was 1 μg/kg.

## Results and Discussion

*A. parasiticus* ATCC 15517 was used as the reference aflatoxin producing strain. The global productivity of aflatoxins B1, B2, G1 and G2 was 25.8 mg/kg on the 8^th^ day and 42.0 mg/kg on the 12^th^ day (Table 1).

The model used for *in vitro* production revealed a higher level of aflatoxins on the 12^th^ day of incubation, 68.0 mg of aflatoxins/kg of humidified cracked corn (Table 3). The specificity of the interaction to a synergic or an antagonistic effect was confirmed for both incubation periods, with the exception of *A. flavus* 1 and *A. glaucus*, that were shown to be synergic on the 8^th^ of incubation and slightly antagonist by the 12^th^ (Table 4). This may signify that there is a specific tendency in the interaction effects of *Aspergilla* strains when they develop concomitantly with a competent aflatoxin producing one, in a particular matrix.

The tendency towards the antagonistic effects seems to be more evident on the 12^th^ day of incubation and the synergic effect was higher, in percentage terms, on the 8^th^ day of incubation (Tables 2 and 3).

The results of the interaction effects of the wild A*spergillus* strains concerning the production level of each of the four aflatoxins by *A. parasiticus* (ATCC 15517), showed special differences with aflatoxin B1, always being present at the highest level, and G2, being the lowest one (Tables 2 and 3).

Synergic activities were detected with strains of *A. candidus, A. clavatus, A. flavus, A. flavipes, A. versicolor* and *A. restritus*, for both incubation periods. The increase of production may be greater then 50% of the reference productivity (3 strains, Table 3). The strains that were revealed to have higher synergistic effects were: *A. flavus* 3 (+ 62.6%), *A. flavipes* (+70.4%) and *A. versicolor* (+ 54.9 %), for the first incubation period. The intermediate metabolic mechanism that may explain this behaviour has not been completely elucidated, although it may be a consequence of some possible intermediate metabolites that can be used by the competent strain to synthesize aflatoxins. The synergism may result from the capacity of the non-toxigenic strains to produce ethylene, or acetate, metabolites that are useful precursors in the biogenesis of aflatoxins [9]. Badii *et al*. [10] reported that precursor metabolites of aflatoxin biosynthesis may justify the synergism between interactive cultures. Another possibility is related to the fact that the non toxigenic strains can also produce sterigmatocystin and *O*-methylsterigmatocystin, chemical precursors of the aflatoxins [11].

Investigations [11] have demonstrated that when these precursors are added to a culture medium in which a toxigenic strain is developing, the level of productivity may increase 3- to 25-fold, compared to reference strain. Synergism may also result from a better efficiency of the competent strain to use the nutrients of the matrix when its metabolism is complementary to that of the challenged strain; the non- toxinogenic strains may have the ability to metabolize each of the nutrients by a different pathway having, for example, a more acentuated proteolitic activity than the toxinogenic strain.

The antagonistic effects were especially evident in the interactive cultures with *A. terreus* (− 47.6%), *A. fumigatus* (− 47.6%) and *A. niger* (− 69.5%), and more marked on the 12^th^ of incubation (Table 4). The decrease of the production was more noticeable for the aflatoxin B1 fraction (Table 3).

Explanations for the antagonistic effects have not been completely elucidated yet, but it may be due to the higher capacity of the challenger strain to more quickly metabolize essential nutrients of the matrix to promote biodegradation of the previously formed aflatoxins. This second hypothesis is in accordance with the fact that antagonist effects were more evident after the longer period of incubation (12 days). A reasonable explanation would invoke the ability of some Aspergilla, especially *A. niger*, to produce organic acids, like citric acid, which leads to a fast pH decrease of the substrate (pH= 3.1 to 3.7), promoting, through this route, an inhibition of growth of the competent aflatoxin producing strain [12].

The antagonistic effects of *A. terreus* are probably related to a fast utilization of nutrients, instead of any specific aflatoxin biodegradation capacity, since the decrease in productivity of aflatoxins B1, B2, G1 and G2 is proportional. Concerning *A. fumigatus*, the substrate conditions were unfavorable for its development, as its optimal growing conditions temperature is above 25° C. The present study shows that the aflatoxin biosynthetic pathway is clearly influenced by the interaction with other moulds that may co-colonize the crops were aflatoxin production occurs. Co-existence and growth of mycobiota on a particular substrate allows for different results in the levels of aflatoxin production capacity of the competent strains. Taking this in consideration, risk assessors should consider biotic interactions when developing models for the characterization of this hazard.

## Figures and Tables

**Table 1.** Data analysis about the productivity of aflatoxins B1, B2, G1 and G2 by *A. parasiticus* (ATCC 15517) in cracked corn, at the 8^th^ and 12^th^ day of incubation.

*A. parasiticus* culture	8^th^ day	12^th^ day
No. assays	14	14
Global Productivity (mg/kg)	25.8	42.0
Standard deviation (SD)	1.42	3.6
Variance	2.02	12.8
Confidence Level (p= 0.05)	0.75	1.88

**Table 2.** Productivity of aflatoxins B1, B2, G1 and G2 in cracked corn, on the 8^th^ day of incubation and the respective cultures.

Cultures	Productivity* (mg/kg)				
	AFB_1_	AFB_2_	AFG_1_	AFG_2_	Total
***A. parasiticus (Ap)***	**10.1**	**8.2**	**3.5**	**3.6**	**25.4**
*Ap* + *A. candidus*	12.0	10.0	8.0	6.0	**36.0**
*Ap* + *A. clavatus*	12.0	10.0	8.0	6.0	**36.0**
*Ap* + *A. flavipes*	18.0	12.0	8.0	6.0	**44.0**
*Ap* + *A. flavus 1*	10.0	9.0	9.0	6.0	**34.0**
*Ap* + *A. flavus 2*	12.0	10.0	9.0	6.0	**37.0**
*Ap* + *A. flavus 3*	18.0	10.0	8.0	6.0	**42.0**
*Ap* + *A. flavus 4*	12.0	10.0	6.0	5.4	**33.4**
*Ap* + *A. flavus 5*	16.0	10.0	6.0	4.0	**36.0**
*Ap* +*A. fumigatus*	6.0	6.0	3.2	2.0	**17.2**
*Ap* + *A. glaucus*	12.0	10.0	6.0	6.0	**34.0**
*Ap* + *A. niger*	3.0	3.0	1.0	1.0	**8.0**
*Ap* + *A. restrictus*	12.0	12.0	6.0	6.0	**36.0**
*Ap* + *A. terreus*	6.0	6.0	3.2	2.0	**17.2**
*Ap*+ *A. versicolor*	12.0	12.0	10.0	6.0	**40.0**

* Average of 2 assays

**Table 3.** Productivity of aflatoxins B1, B2, G1 and G2 in cracked corn, on the 12^th^ day of incubation and respective cultures.

Cultures	Productivity* (mg/kg)				
	AFB_1_	AFB_2_	AFG_1_	AFG_2_	Total
***A. parasiticus (Ap)***	**18.0**	**13.3**	**7.0**	**3.7**	**42.0**
*Ap* + *A. candidus*	18.0	16.0	10.0	10.0	**54.0**
*Ap* + *A. clavatus*	18.0	18.0	12.0	12.0	**60.0**
*Ap* + *A. flavipes*	30.0	20.0	12.0	6.0	**68.0**
*Ap* + *A. flavus 1*	18.0	6.0	9.0	6.0	**39.0**
*Ap* + *A. flavus 2*	20.0	10.0	9.0	6.0	**45.0**
*Ap* + *A. flavus 3*	24.0	10.0	12.0	6.0	**52.0**
*Ap* + *A. flavus 4*	20.0	18.0	12.0	6.0	**56.0**
*Ap* + *A. flavus 5*	20.0	16.0	9.0	8.0	**53.0**
*Ap* +*A. fumigatus*	8.0	8.0	4.0	2.0	**22.0**
*Ap* + *A. glaucus*	16.0	12.0	4.0	3.0	**35.0**
*Ap* + *A. niger*	6.0	4.0	1.4	1.4	**12.8**
*Ap* + *A. restrictus*	20.0	16.0	10.0	6.0	**52.0**
*Ap* + *A. terreus*	8.0	8.0	4.0	2.0	**22.0**
*Ap*+ *A. versicolor*	18.0	16.0	10.0	10.0	**54.0**

*Average of 2 assays

**Table 4.** Productivity deviation ratio (%) by *Aspergillus* strains relative to the reference (*A. parasiticus*) global productivity of aflatoxins B1, B2, G1 and G2.

Cultures	Productivity deviation ratio (%) by *Aspergillus* strains relative to the testimony		Interactions
	8^th^ day	12^th^ day	
*Ap* + *A. candidus*	39.4	28.6	Synergic
*Ap* + *A. clavatus*	39.4	42.9	Synergic
*Ap* + *A. flavipes*	70.4	62.0	Synergic
*Ap* + *A. flavus 1*	31.6	−7.1	Synergic/Antagonist
*Ap* + *A. flavus 2*	43.3	7.2	Synergic
*Ap* + *A. flavus 3*	62.6	23.9	Synergic
*Ap* + *A. flavus 4*	29.3	33.4	Synergic
*Ap* + *A. flavus 5*	39.4	26.2	Synergic
*Ap* +*A. fumigatus*	−33.4	−47.6	Antagonist
*Ap* + *A. glaucus*	31.6	−16.6	Synergic/Antagonist
*Ap* + *A. niger*	−69.0	−69.5	Antagonist
*Ap* + *A. restrictus*	39.4	23.9	Synergic
*Ap* + *A. terreus*	−33.4	−47.6	Antagonist
*Ap*+ *A. versicolor*	54.9	28.6	Synergic

## References

[1] Betina V (1989). Mycotoxins, Chemical, Biological and Environmental Aspects.

[2] Hsieh DPH, Krogh P (1987). Mycotoxins in food. Mode of action of mycotoxins.

[3] Domsch KH, Gams W, Anderson TH (1980). Compendium of Soil Fungi.

[4] Maggon K, Gupta S, Venkitasubranian T (1977). Biosynthesis of Aflatoxins. Bacteriol Rev.

[5] European Comission (2007). DGSANCO The Rapid Alert System for Food and Feed (RASFF) Annual Report 2006.

[6] Martins ML (1989). Capacidade de produção de Aflatoxinas por Aspergillus flavus em substratos naturais. Rep Trab, LNIV.

[7] Martins HM, Bernardo F, Martins ML (1999). Produção de Aflatoxinas “in vitro”. Rev Port Ciên Veter.

[8] Stroka J, Anklam E, Jorissen U, Gilbert J (2000). Immunoaffinity column cleanup with liquid chromatography using post-column brominatation for determination of aflatoxins in peanut butter, pistachio paste, fig paste, and paprika powder: collaborative study. JAOAC Int.

[9] Sharma A, Dessai SRP, Nad Karni GB (1985). Possible implications of reciprocity between ethylene and Aflatoxin Biogenesis in A. flavus and A. parasiticus. Appl Environm Microbiol.

[10] Badii F, Moss O, Wilson K (1986). The effect of Sodium Biselenite on the growth and Aflatoxin production of A. parasiticus and the growth of other Aspergilli. Lett Appl Microbiol.

[11] Pro ML, Moreno MA, Suarez G (1991). Transformation of sterigmotocystin and o–metal sterigmatocystin by Aflatoxigenic and nonaflatoxigenic field isolates of the A. flavus. Mycophathology.

[12] Horn BW, Wicklow DT (1983). Factors influencing the inhibition of aflatoxin production in corn by Aspergillus niger. Can J Microbiol.

